# High-Iron Consumption Impairs Growth and Causes Copper-Deficiency Anemia in Weanling Sprague-Dawley Rats

**DOI:** 10.1371/journal.pone.0161033

**Published:** 2016-08-18

**Authors:** Jung-Heun Ha, Caglar Doguer, Xiaoyu Wang, Shireen R. Flores, James F. Collins

**Affiliations:** Food Science and Human Nutrition Department, University of Florida, Gainesville, Florida, United States of America; Lady Davis Institute for Medical Research, CANADA

## Abstract

Iron-copper interactions were described decades ago; however, molecular mechanisms linking the two essential minerals remain largely undefined. Investigations in humans and other mammals noted that copper levels increase in the intestinal mucosa, liver and blood during iron deficiency, tissues all important for iron homeostasis. The current study was undertaken to test the hypothesis that dietary copper influences iron homeostasis during iron deficiency and iron overload. We thus fed weanling, male Sprague-Dawley rats (n = 6-11/group) AIN-93G-based diets containing high (~8800 ppm), adequate (~80) or low (~11) iron in combination with high (~183), adequate (~8) or low (~0.9) copper for 5 weeks. Subsequently, the iron- and copper-related phenotype of the rats was assessed. Rats fed the low-iron diets grew slower than controls, with changes in dietary copper not further influencing growth. Unexpectedly, however, high-iron (HFe) feeding also impaired growth. Furthermore, consumption of the HFe diet caused cardiac hypertrophy, anemia, low serum and tissue copper levels and decreased circulating ceruloplasmin activity. Intriguingly, these physiologic perturbations were prevented by adding extra copper to the HFe diet. Furthermore, higher copper levels in the HFe diet increased serum nonheme iron concentration and transferrin saturation, exacerbated hepatic nonheme iron loading and attenuated splenic nonheme iron accumulation. Moreover, serum erythropoietin levels, and splenic erythroferrone and hepatic hepcidin mRNA levels were altered by the dietary treatments in unanticipated ways, providing insight into how iron and copper influence expression of these hormones. We conclude that high-iron feeding of weanling rats causes systemic copper deficiency, and further, that copper influences the iron-overload phenotype.

## Introduction

Iron is an essential trace element that is required for oxygen transport and storage, energy metabolism, antioxidant function and DNA synthesis. Abnormal iron status, as seen in iron deficiency and iron overload, perturbs normal physiology. Copper is also an essential nutrient for humans, being involved in energy production, connective tissue formation and neurotransmission. Copper, like iron, is required for normal erythropoiesis; copper deficiency causes an iron-deficiency-like anemia [1]. Moreover, copper homeostasis is closely linked with iron metabolism, since iron and copper have similar physiochemical and toxicological properties. Physiologically-relevant iron-copper interactions were first described in the mid-1800s, when chlorosis or the “greening sickness” was abundant in young women of industrial Europe [2]. Although specific clinical information is lacking, chlorosis likely resulted from iron-deficiency anemia (IDA) [1], a condition which was, and still is, common in this demographic group. Women who worked in copper factories were, however, protected from chlorosis [2], suggesting that copper positively influences iron homeostasis [1].

Iron-copper interactions in biological systems may be attributed to their positive charges, similar atomic radii, and common metabolic fates. For example, dietary iron and copper are both absorbed in the proximal small intestine [1]. Also, iron and copper must be reduced before uptake into enterocytes and further, both metals are oxidized after (or concurrent with) export into the interstitial fluids (enzymatic iron oxidation may occur while copper oxidation is likely spontaneous). Moreover, both metals are involved in redox chemistry in which they function as enzyme cofactors, and both can be toxic when in excess. Furthermore, a reciprocal relationship between iron and copper has been established in some tissues. For example, copper accumulates in the liver during iron deficiency, and iron accumulates during copper deficiency [1, 2]. Copper levels also increase in the intestinal mucosa and blood during iron deprivation [2, 3]. Despite these intriguing past observations, the molecular bases of physiologically-relevant iron-copper interactions are yet to be elucidated in detail. The aim of this investigation was thus to provide additional, novel insight into the interplay between iron and copper.

We have been investigating how copper influences intestinal iron absorption during iron deficiency for the past decade. It was noted that an enterocyte copper transporter, copper-transporting ATPase 1 (Atp7a), was strongly induced during iron deficiency in rats [3, 4] and mice [5]. Additional experimentation demonstrated that the mechanism of *Atp7a* induction was via a hypoxia-inducible transcription factor (Hif2α) [6, 7]. Importantly, this transcriptional mechanism is also invoked to increase expression of the intestinal iron importer (divalent metal-ion transporter 1 [Dmt1]), a brush-border membrane (BBM) ferrireductase (duodenal cytochrome b [Dcytb]), and the basolateral membrane (BLM) iron exporter (ferroportin 1 [Fpn1]). Moreover, it was suggested that the principle intestinal iron importer, Dmt1, could transport copper during iron deficiency [8]. In the current investigation, we sought to broaden our experimental approach by testing the hypothesis that dietary copper will influence iron metabolism during iron deficiency and iron overload (both being conditions that cause significant homeostatic perturbations in humans). The study design was to feed male, weanling, Sprague-Dawley rats one of 9 different diets, varying only in iron and copper content (low, adequate or high), for 5 weeks. After the dietary treatments, iron- and copper-related phenotypical parameters were analyzed to assess the impact of variable copper levels on iron homeostasis.

## Materials and Methods

### Animal Experiments

All animal studies were approved by the University of Florida IACUC. Three-week-old, male Sprague-Dawley rats (Harlan; Indianapolis, IN) were housed in stainless steel overhanging, wire mesh-bottom cages for 5 weeks until sacrifice. The 5 week time period was selected based upon our prior experience working with SD rats and related to the time point when significant iron deficiency has been noted (as reported in our previously published studies [3, 4, 9, 10]). The rats had *ad libitum* access to food and purified water. Diets were fabricated based on the AIN-93G formulation [11, 12] (Dyets Inc.; Bethlehem, PA) and contained high (HFe), adequate (ADFe) or low (LFe) iron in combination with high (HCu), adequate (AdCu) or low (LCu) copper (Tables 1–3). We increased the amount of iron in the AdFe diet (from 50 ppm to 80) to ensure normal growth of these weanling rats. The HFe diets were modeled after published studies [13, 14]. The HCu diets contained ~20 times more copper than the adequate level. The rationale for this was that we anticipated that this amount of dietary copper would increase copper absorption yet be well below a toxic amount. Moreover, all diets contained extra sucrose (100 g/kg), as high carbonyl iron diets are otherwise unpalatable. All LFe and AdFe diets were isocaloric (3760 kcal/kg); however, the HFe diets contained slightly less energy (3724 kcal/kg; <1% less) since 10 g/kg of carbonyl iron was added (in place of a small amount of corn starch). Furthermore, animals were weighed weekly and food consumption was estimated by weighing the amount of food provided daily to each cage of rats. Animals were sacrificed by thoracotomy after CO_2_ narcosis.

**Table 1:** Iron and Copper Concentrations in Experimental Diets.

Diet	Fe (ppm)^≠^	Cu (ppm) ^≠^
LFe/LCu*	12.0	0.83
LFe/AdCu	8.84	6.65
LFe/HCu	12.4	182
AdFe/LCu	93.7	0.92
AdFe/AdCu	71.9	8.96
AdFe/HCu	71.8	183
HFe/LCu	9036	0.94
HFe/AdCu	8707	9.18
HFe/HCu	8718	184

* H, high; Ad, adequate; L, low

^≠^ determined by ICP/MS

**Table 2:** Constant Ingredients in the 9 Experimental Diets.

Ingredient	Amount (g/kg)
Casein	200
Sucrose	100
Soybean oil	70
t-Butyhydroquinone	0.014
Dyetose	132
Cellulose (micro)	50
Mineral Mix	35
Vitamin Mix	10
Choline Bitartrate	2.5
L-Cystine	3

**Table 3:** Variable Ingredients in the 9 Experimental Diets.

Ingredient	LFe/LCu	LFe/AdCu	LFe/HCu	AdFe/LCu	AdFe/AdCu	AdFe/HCu	HFe/LCu	HFe/AdCu	HFe/HCu
Cornstarch (g/kg)	397.486	397.486	397.486	397.486	397.486	397.486	387.486	387.486	387.486
Fe Premix (10 mg/g)	1	1	1	8	8	8	-	-	-
Carbonyl Fe (g/kg)	-	-	-	-	-	-	10	10	10
Cu Premix (1 mg/g)	0.5	-	-	0.5	-	-	0.5	-	-
Cu Premix (5 mg/g)	-	1.6	40	-	1.6	40	-	1.6	40
kcal/kg	3760	3760	3760	3760	3760	3760	3724	3724	3724

### Determination of Iron and Copper Status

Hemoglobin (Hb) and hematocrit (Hct) levels were determined as described previously [5], using standard protocols. Liver samples were digested in acid solution (3 mol/L HCl, 10% trichloroacetic acid) and nonheme iron levels were determined, as previously described [15]. Serum nonheme iron levels were quantified using a common colorimetric method [16]. For measurement of total iron-binding capacity (TIBC), a previously described colorimetric method was used [17, 18]. Transferrin (Tf) saturation was subsequently calculated as: serum iron/ TIBC x 100. Serum erythropoietin (Epo) levels were determined by ELISA (cat # LS-F10511; LifeSpan BioSciences Inc.; Seattle, WA). To assess serum ceruloplasmin (Cp) activity, a *p*PD assay, which measures the amine oxidase activity of Cp, was performed according to a previously reported method [19, 20].

### qRT-PCR

Total cellular RNA was isolated with RNAzol® RT reagent (Molecular Research Center Inc.; Cincinnati, OH) as previously described [21]. SYBR-Green qRT-PCR was performed according to a well-established protocol [9]. Oligonucleotide primers (Table 4) spanned large introns to avoid amplification from genomic DNA. Standard-curve reactions validated each primer pair, and melt curves routinely showed single amplicons. Expression of experimental genes was normalized to expression of cyclophilin (which did not significantly vary between groups).

**Table 4:** qRT-PCR Primer Sequences.

Primer	Forward	Reverse
Cyclophilin	5’-CTTGCTGCAATGGTCAACC-3’	5’-TGCTGTCTTTGGAACTTTGTCTGC-3’
Bmp6	5’-CTTACGACAAGCAGCCCTTCATG-3’	5’-AGCTGTTTTTAACTCACTGCTGTTGTA-3’
Epo	5’-AGTCGCGTTCTGGAGAGGTA-3’	5’-ACTTTGGTATCTGGGACGGTAA-3’
Erfe	5’-ACTCACCAAGCAGCCAAGAA-3’	5’-TTCTCCAGCCCCATCACAGT-3’
Il-6	5’-GCCCTTCAGGAACAGCTATG-3’	5’-ACTGGTCTGTTGTGGGTGGT-3’

Forward and reverse primers used for PCR analysis of gene expression are listed.

### Statistical Analysis

The homogeneity of variances was determined by the Fligner-Killeen test. If there was not homogeneity of variance in the data set, then data were transformed as a log_10_ scale prior to performing the statistical analyses. All statistical analyses were thus performed on data with equal variances. All results are expressed as means ± SDs or Box-and-Whisker plots except correlation data. Fligner-Killeen tests were performed in R (version 3.3.2) and the remaining analyses were performed using GraphPad (version 6.0.4 for Windows). The trends in data were analyzed using a 2-factor ANOVA test. If this analysis showed significant iron X copper interactions (*p*<0.05), Tukey’s multiple comparisons *post hoc* test was utilized to identify groups which varied significantly for a given parameter. A summary of all statistical comparisons is provided in Table 5 (iron main effect, copper main effect and iron X copper interactions); select comparisons are provided in the figure legends. Furthermore, Pearson product-moment correlation coefficient (r) was calculated to clarify relationships between two variables.

**Table 5:** Statistical Summary.

Parameter	Fe main effect	Cu main effect	Fe x Cu interaction
Growth rate	**** *p*<0.0001	**** *p*<0.0001	*** *p* = 0.0002
Final body weight	**** *p*<0.0001	** *p* = 0.0055	*** *p* = 0.0001
Liver/Body weight	**** *p*<0.0001	ns *p* = 0. 2303	* *p* = 0.0139
Heart/Body weight	**** *p*<0.0001	**** *p*<0.0001	** *p* = 0.0011
Spleen/Body weight	*** *p* = 0.0002	ns *p* = 0.0688	**** *p*<0.0001
Kidney/Body weight	ns *p* = 0.6499	ns *p* = 0.6246	ns *p* = 0.2177
Hb	**** *p*<0.0001	**** *p*<0.0001	**** *p*<0.0001
Hct (%)	**** *p*<0.0001	**** *p*<0.0001	**** *p*<0.0001
Nonheme serum Fe	**** *p*<0.0001	*** *p* = 0.0001	**** *p*<0.0001
Nonheme liver Fe^≠^	**** *p*<0.0001	** *p* = 0.0064	** *p* = 0.0026
Nonheme splenic Fe^≠^	**** *p*<0.0001	** *p* = 0.0064	** *p* = 0.0026
TIBC	**** *p*<0.0001	ns *p* = 0.1559	*** *p* = 0.0007
Tf saturation (%)	**** *p*<0.0001	**** *p*<0.0001	*** *p* = 0.0006
Epo (mRNA)^≠^	**** *p*<0.0001	**** *p*<0.0001	**** *p*<0.0001
Epo (protein)^≠^	**** *p*<0.0001	**** *p*<0.0001	**** *p*<0.0001
Hepc (mRNA)^≠^	**** *p*<0.0001	ns *p* = 0.5728	*** *p* = 0.0010
Erfe (mRNA)	**** *p*<0.0001	*** *p* = 0.0002	**** *p*<0.0001
Cp activity	**** *p*<0.0001	**** *p*<0.0001	**** *p*<0.0001
Bone Fe	**** *p*<0.0001	ns *p* = 0.6337	ns *p* = 0.1918
Enterocyte Fe	*** *p* = 0.0003	ns *p* = 0.2844	ns *p* = 0.4764
Liver Fe^≠^	**** *p*<0.0001	ns *p* = 0.6249	** *p* = 0.0017
Heart Fe	ns *p* = 0.1825	ns *p* = 0.7213	ns *p* = 0.0934
Kidney Fe	**** *p*<0.0001	** *p* = 0.0068	** *p* = 0.0040
Serum Fe	**** *p*<0.0001	ns *p* = 0.3135	*** *p* = 0.0002
Bone Cu	** *p* = 0.0019	**** *p*<0.0001	ns *p* = 0.5927
Enterocyte Cu	ns *p* = 0.0884	**** *p*<0.0001	** *p* = 0.0019
Liver Cu	**** *p*<0.0001	**** *p*<0.0001	**** *p*<0.0001
Heart Cu	**** *p*<0.0001	**** *p*<0.0001	**** *p*<0.0001
Kidney Cu	*** *p* = 0.0001	**** *p*<0.0001	ns *p* = 0.1579
Serum Cu	* *p* = 0.0445	**** *p*<0.0001	** *p* = 0.0016
Est. av. calorie intake	* *p* = 0.0163	ns *p* = 0.0734	ns *p* = 0.5834

ns = not significant

* *p*<0.005

** *p*<0.001

*** *p*<0.0005

**** *p*<0.0001

^≠^ Statistical analysis performed from log_10_ transformed data.

## Results

### Growth Rates and Organ Weights Differed Among Experimental Groups

**Fig 1:**
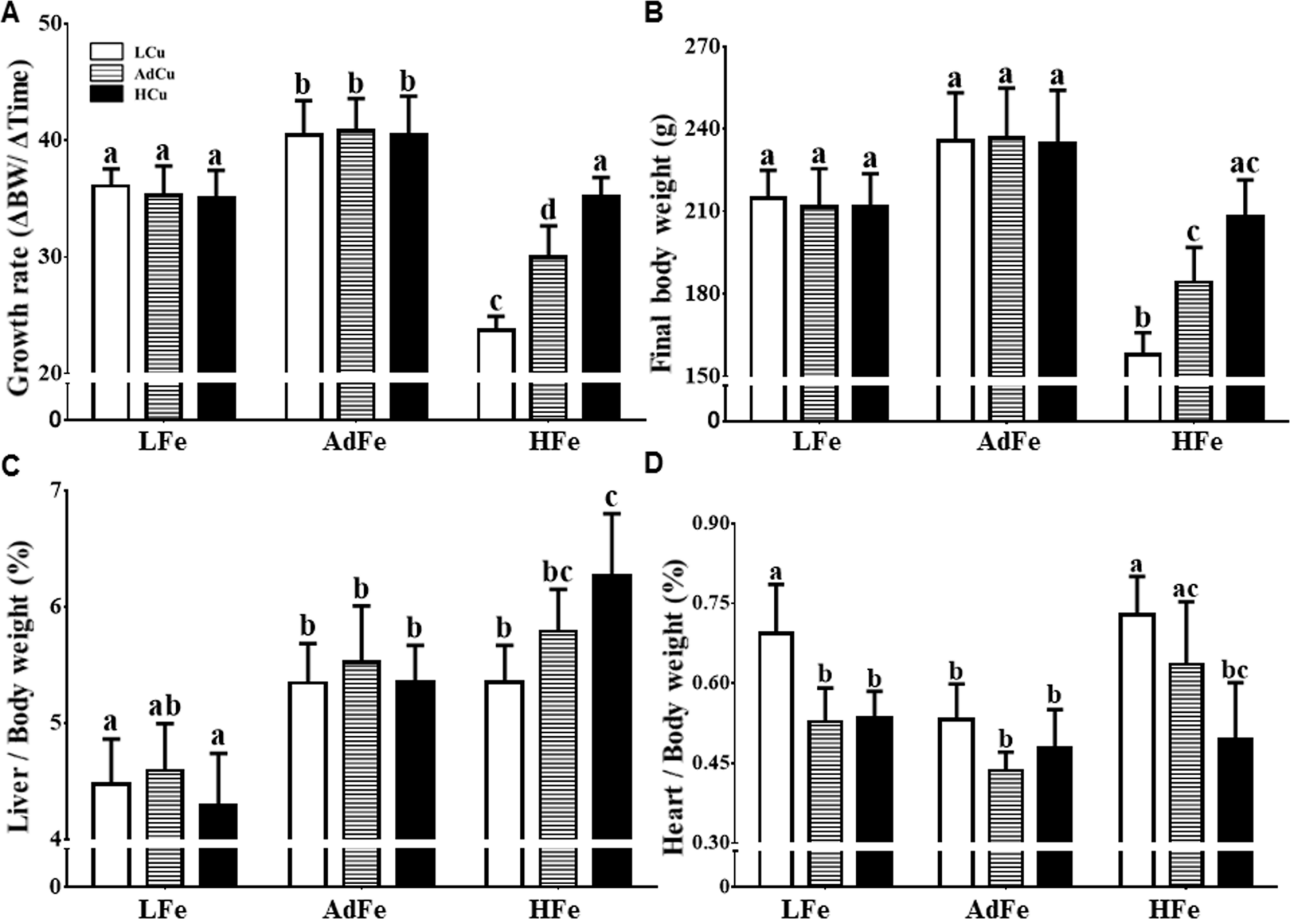
High-iron feeding impaired growth and caused cardiac hypertrophy. Weanling rats were fed one of 9 diets differing only in iron and copper content for 5 weeks *ad libitum*. Rats were weighed weekly, and growth rates were calculated (**A**). Final body weights (**B**), and liver (**C**) and heart (**D**) weights at sacrifice are also shown. Organ weights were normalized by body weight. Values are means ± SDs. Labeled means without a common letter differ (*p*<0.05). Animal numbers were as follows: LFe/LCu, n = 9; LFe/AdCu and LFe/HCu, n = 6; AdFe/AdCu, n = 11; and all others, n = 10. These same n values apply to all data presented in this manuscript (which will not be repeated in subsequent figure legends). Abbreviations: L, low; Ad, adequate; H, high. 2-way ANOVA factor analysis results are as follows: growth rate (Fe: *p*<0.0001; Cu: *p*<0.0001; Fe X Cu: *p*<0.001); final body weight (Fe: *p*<0.0001; Cu: *p*<0.01; Fe X Cu: *p*<0.001); liver weights (Fe: *p*<0.0001; Cu: ns; Fe X Cu: *p*<0.05); and heart weights (Fe: *p*<0.0001; Cu: *p*<0.0001; Fe X Cu: *p*<0.01). ns, not significant.

Rats consuming the LFe diets grew slower than controls (i.e. the AdFe/AdCu group), irrespective of copper content. Unexpectedly, rats fed the HFe diets also showed a significant reduction in growth rate and final body weight, but increasing copper content (from low to high) progressively restored these parameters (Fig 1A and 1B). Alterations in growth were probably not the result of changes in energy intake as the amount of food provided to the different experimental groups was similar (Table 6). Moreover, liver weights were generally lower in the LFe groups, while consumption of the HFe/HCu diet increased liver weights (as compared to all AdFe and the HFe/LCu groups) (Fig 1C). Heart weights were higher in the LFe/LCu, HFe/LCu and HFe/Ad Cu groups (Fig 1D); adding extra copper to these diets, however, prevented cardiac hypertrophy. Spleens were larger only in rats consuming the LFe/LCu diet, while kidney weights did not vary significantly among groups (Table 7). In sum, these data suggest that high-iron feeding impairs copper homeostasis, given that cardiac hypertrophy is a hallmark of severe copper deficiency and that this was prevented by higher copper intake. Further supporting this possibility are the noted anemia and growth impairment (in the absence of iron deficiency), which also typify copper deprivation.

**Table 6:** Estimated Average Daily Calorie Intake.

Dietary Gro	LFe/LCu	LFe/AdCu	LFe/HCu	AdFe/LCu	AdFe/AdCu	AdFe/HCu	HFe/LCu	HFe/AdCu	HFe/HCu
kcal/rat/d	25.6 ± 2.11* (9)^≠^	24.6 ± 2.83 (6)	25.4 ± 1.33 (6)	27.6 ± 1.40 (6)	25.6 ± 2.91 (6)	28.7 ± 1.47 (6)	23.3 ± 0.86 (6)	23.7 ± 1.58 (6)	26.4 ± 2.04 (6)

* Values are means ± SDs.

^≠^ Numbers in parentheses indicate n values.

**Table 7:** Relative Spleen and Kidney Weights.

% of BW	LFe/LCu	LFe/AdCu	LFe/HCu	AdFe/LCu	AdFe/AdCu	AdFe/HCu	HFe/LCu	HFe/AdCu	HFe/HCu
Spleen	0.52 ± 0.06^a^* (8)^≠^	0.32 ± 0.03^b^ (6)	0.34 ± 0.04^b^ (6)	0.28 ± 0.05^b^ (6)	0.33 ± 0.02^b^ (6)	0.36 ± 0.06^b^ (6)	0.31 ± 0.12^b^ (6)	0.33 ± 0.06^b^ (6)	0.39 ± 0.11^b^ (6)
Kidney	0.51 ± 0.10 (8)	0.46 ± 0.02 (6)	0.45 ± 0.05 (6)	0.43 ± 0.04 (6)	0.44 ± 0.04 (6)	0.48 ± 0.05 (6)	0.51 ± 0.10 (6)	0.51 ± 0.04 (6)	0.50 ± 0.04 (6)

* Values are means ± SDs. Labeled means without a common letter differ, p<0.05 (2-factor ANOVA).

^≠^ Numbers in parentheses indicate n values.

### Low- and High-Iron Consumption Altered Hematological Parameters

**Fig 2:**
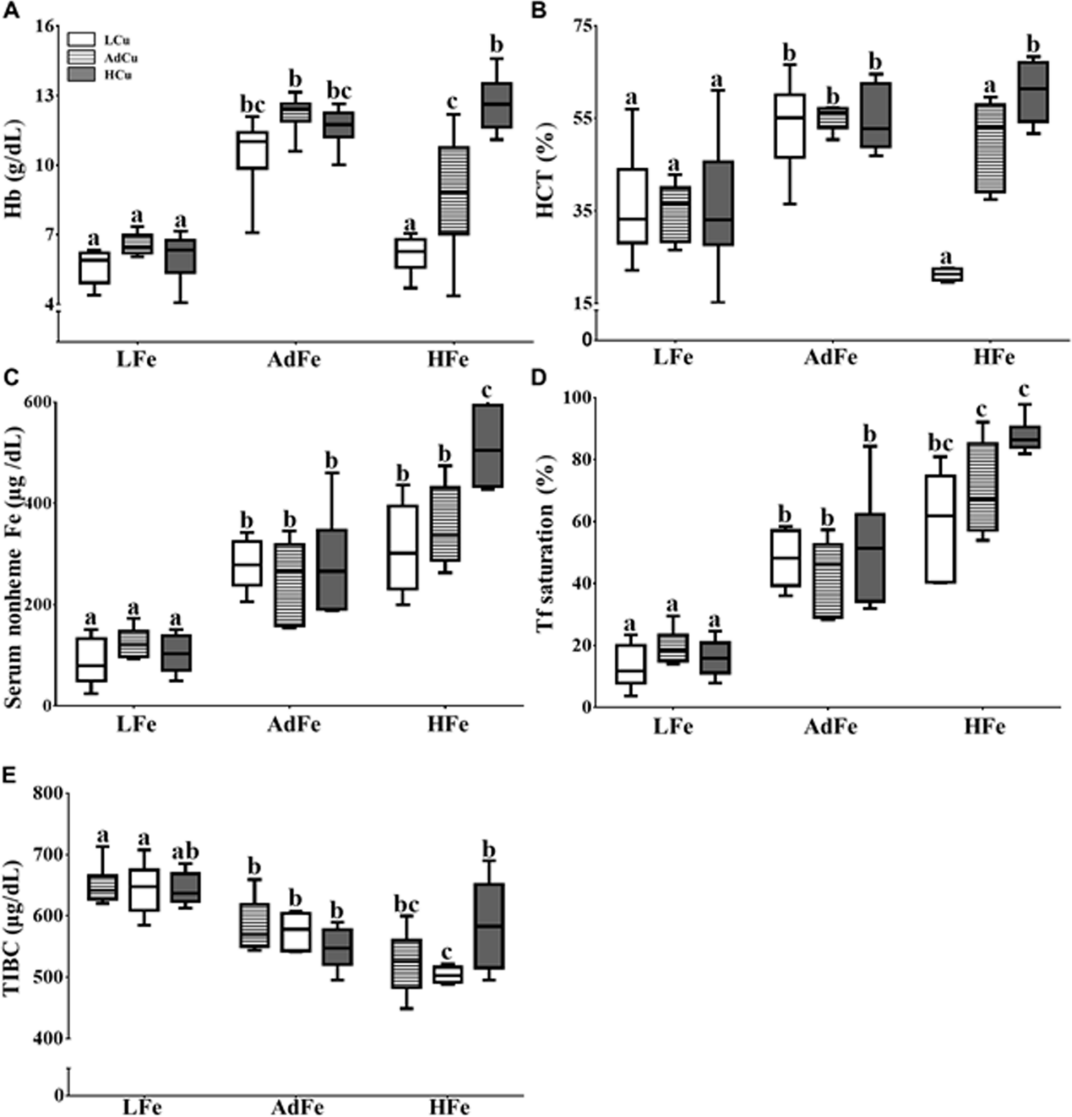
Consumption of the low- and high-iron diets altered hematological parameters. Hemoglobin (Hb) (**A**) and hematocrit (Hct) (**B**) were determined from whole blood collected from experimental animals at sacrifice. Serum nonheme iron (**C**), serum transferrin saturation (**D**) and total iron-binding capacity (TIBC) (**E**) were also quantified. Labeled means without a common letter differ (*p*<0.05). n values and abbreviations used are the same as in Fig 1. The Box-and-Whisker plots indicate the following: the minimum value (the lower whisker), the lower quartile, the median, the upper quartile and the maximum value (the upper whisker). 2-way ANOVA factor analysis results are as follows: hemoglobin (Fe: *p*<0.0001; Cu: *p*<0.0001; Fe X Cu: *p*<0.0001); hematocrit (Fe: *p*<0.0001; Cu: *p*<0.0001; Fe X Cu: *p*<0.0001); serum nonheme iron (Fe: *p*<0.0001; Cu: *p*<0.001; Fe X Cu: *p*<0.0001); serum transferrin saturation (Fe: *p*<0.0001; Cu: *p*<0.0001; Fe X Cu: *p*<0.001) and TIBC (Fe: *p*<0.0001; Cu: ns; Fe X Cu: *p*<0.001). ns, not significant.

Hb levels were depressed in rats consuming the LFe diets with copper content not having any affect (Fig 2A). Hb levels were also lower in rats fed the HFe/LCu and HFe/AdCu diets, but consumption of the HFe/HCu diet prevented deficits in Hb. A similar trend was noted in Hct levels (Fig 2B). Moreover, nonheme serum iron was low in the LFe groups, while HFe feeding did not alter this parameter except for in the HFe/HCu group, in which it was significantly increased (Fig 2C). Tf saturation was also depressed in the LFe groups, and values were increased significantly in the HFe/AdCu and HFe/HCu groups (Fig 2D). Furthermore, TIBC trended higher in the LFe groups (Fig 2E). These observations further support the postulate that high-iron feeding perturbs copper homoeostasis, since copper deficiency causes an iron deficiency-like anemia. Prevention of the anemia by increasing the copper content of the HFe diet is also congruent with this supposition.

### Renal Epo Expression Was Induced by Copper Deprivation in Iron-Deficient and Iron-Loaded Rats

**Fig 3:**
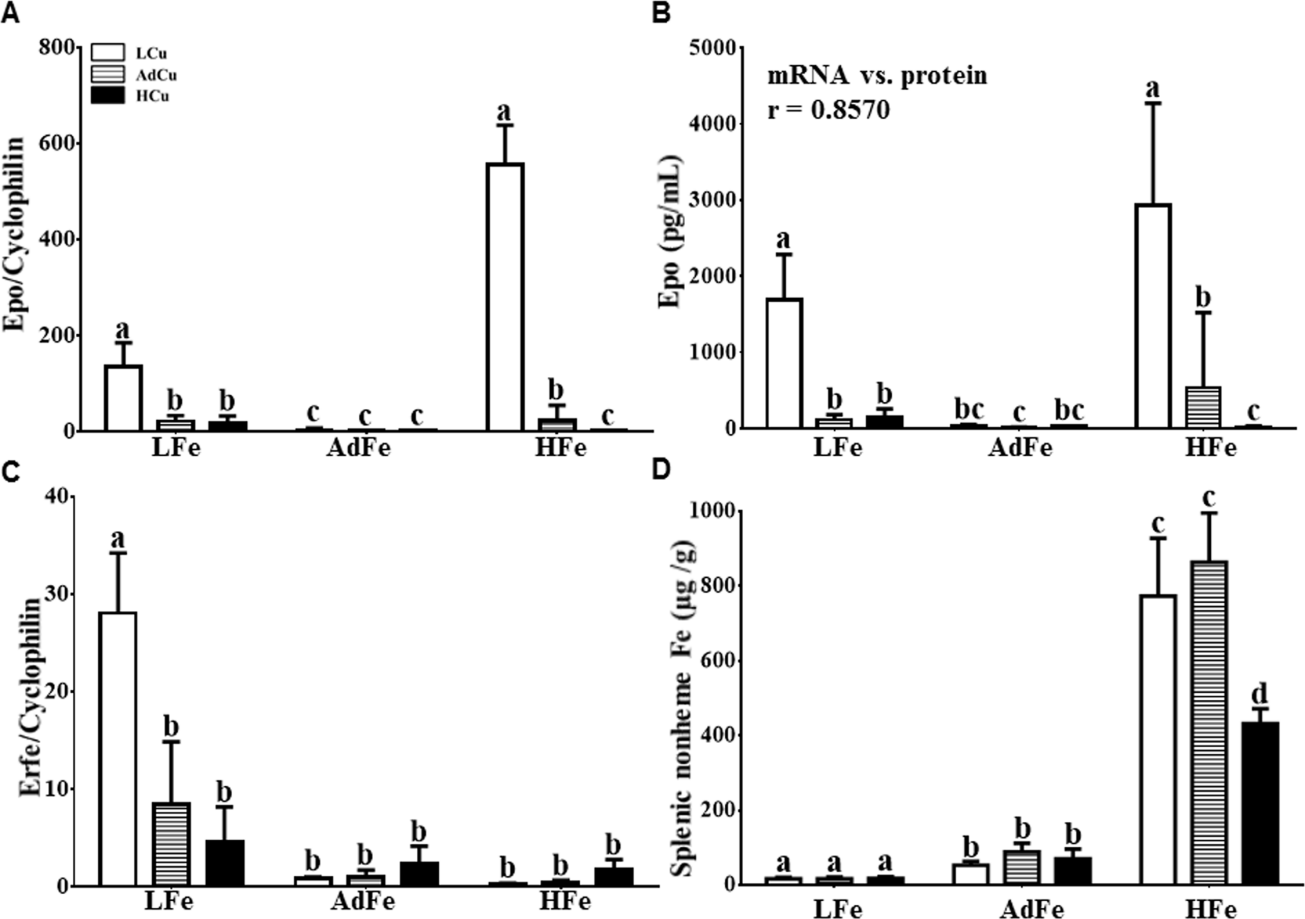
Renal Epo and splenic Erfe levels increased in rats consuming the LFe/LCu diet. Renal Epo mRNA (**A**) and serum Epo protein levels (**B**) were assessed in experimental rats. The Pearson product-moment correlation coefficient (r) comparing these 2 parameters is noted in the inset of panel B (*p*<0.0001). Splenic Erfe mRNA expression was quantified by qRT-PCR (**C**) and splenic nonheme iron levels (**D**) were measured using a commonly used technique. Labeled means without a common letter differ (*p*<0.05). Values are means ± SDs. n values and abbreviations used are the same as in Fig 1. Data for Epo mRNA and protein expression and splenic nonheme iron concentration were log_10_ transformed prior to running statistical analyses due to large variations in values. For ease of interpretation, we have, however, presented the non-transformed data in the figure. 2-way ANOVA factor analysis results are as follows: renal Epo mRNA (Fe: *p*<0.0001; Cu: *p*<0.0001; Fe X Cu: *p*<0.0001); serum Epo protein (Fe: *p*<0.0001; Cu: *p*<0.0001; Fe X Cu: *p*<0.0001); splenic Erfe (Fe: *p*<0.0001; Cu: *p*<0.001; Fe X Cu: *p*<0.0001); and splenic nonheme iron (Fe: *p*<0.0001; Cu: *p*<0.01; Fe X Cu: *p*<0.01).

Since hematological parameters were altered, unexpectedly, in rats consuming the HFe diets, we next assessed levels of the erythroid hormone, erythropoietin (Epo). Renal Epo mRNA expression and serum Epo protein levels were significantly increased in rats consuming the LFe/LCu and HFe/LCu diets (Fig 3A and 3B). Moreover, a strong correlation between renal Epo mRNA and serum Epo protein levels was noted (Fig 3B, inset). Increased Epo levels only in rats consuming the LFe/LCu and HFe/LCu diets suggests that copper deprivation increases Epo expression, independent of hypoxia or anemia, since some anemic, presumably hypoxic, rats (e.g. in the LFe/AdCu, LFe/HCu and HFe/AdCu groups) did not show such dramatic increases in Epo expression.

### The Erythroid Iron Regulator, Erfe, Was Induced by Copper Deprivation in the Spleens of Iron-Deficient Rats

Recently, an erythropoietic stress-related hormone, erythroferrone ([Erfe]) [22], was discovered. Erfe is expressed in developing erythrocytes and spleen (which is an erythropoietic organ in rodents). Erfe was reported to be induced by circulating Epo and it functions to suppress hepatic *Hamp* (the gene encoding hepcidin [Hepc]) expression. Given that serum Epo protein levels increased in some of our experimental rats, we next assessed splenic Erfe mRNA expression. Quantification of mRNA levels is of relevance, since Erfe is regulated at the transcript level by erythropoietic stress [23]. Erfe expression was dramatically increased only in rats consuming the LFe/LCu diet (Fig 3C). Surprisingly, Erfe expression was, however, not increased in the HFe/LCu group, despite significant anemia/hypoxia and strong induction of Epo expression in these animals. Therefore, since Erfe was only induced in anemic rats in the setting of low splenic iron (Fig 3D), we speculate that induction of Erfe expression by Epo may be inhibited by iron accumulation. This would be a logical supposition since suppression of Hepc expression during iron loading would only exacerbate tissue iron accumulation. This is also consistent with the noted suppression of Hepc expression (as described below) in only the low iron-fed rats.

### Hepatic Nonheme Iron Loading Increased in the HFe/HCu Group

**Fig 4:**
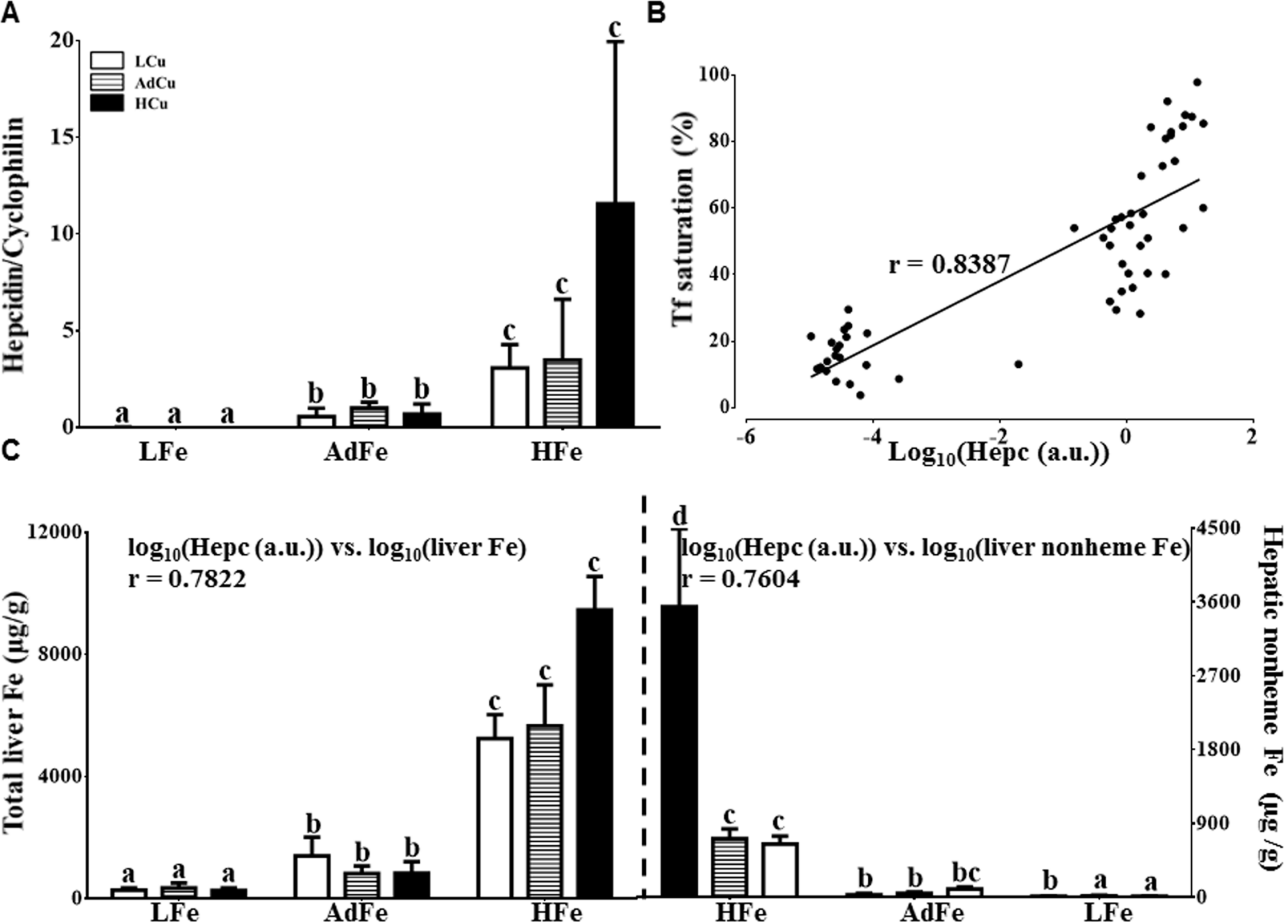
Extra copper in the HFe diets did not influence Hepc mRNA expression but it accentuated hepatic nonheme iron loading. Hepc mRNA expression was quantified in experimental rats (**A**), and the relationship between Hepc mRNA expression (log_10_) and Tf saturation was estimated by calculating Pearson product-moment correlation (**B**). The line of best fit is shown along with the correlation coefficient (r) (*p*<0.0001). Hepatic total (**C, left side**) and nonheme (**C, right side**) iron was also measured. Note that the 2 halves of panel **C** are mirror images with respect to the order of the experimental groups. Correlations were also calculated between Hepc mRNA expression (log_10_) and liver iron levels (log_10_) (r values are shown as insets) (**C**). Labeled means without a common letter differ (*p*<0.05). Values are means ± SDs. n values and abbreviations used are the same as in Fig 1. Data for Hepc mRNA and hepatic total and nonheme iron concentrations were log_10_ transformed prior to running statistical analysis due to large variations in values. For ease of interpretation, we have, however, presented the non-transformed data in the figure. a.u., arbitrary units. 2-way ANOVA factor analysis results are as follows: Hepc (Fe: *p*<0.0001; Cu: ns; Fe X Cu: *p*<0.001); total liver iron (Fe: *p*<0.0001; Cu: ns; Fe X Cu: *p*<0.01); and hepatic nonheme iron (Fe: *p*<0.0001; Cu: *p*<0.01; Fe X Cu: *p*<0.01). ns, not significant.

Hepc is a liver-derived, peptide hormone that is considered the master regulator of iron homeostasis. Given that *Hamp* expression is controlled predominantly at the level of transcription, we next quantified hepatic Hepc mRNA levels. As expected, Hepc mRNA expression was essentially nil in all rats consuming the LFe diets (Fig 4A). Consumption of the HFe diet increased Hepc mRNA expression, with higher copper content leading to a trend towards a more dramatic increase. Since one driver of *Hamp* gene expression is serum Tf saturation [24, 25], Pearson’s test was utilized to relate Hepc mRNA expression to Tf saturation. Results showed a strong correlation (Fig 4B). Moreover, hepatic iron levels paralleled changes in Hepc mRNA expression (Fig 4C). The most significant iron loading was noted in rats consuming the HFe diet with extra copper, which coincidently, is the same group that had the highest Hepc expression. There was also a strong correlation between Hepc mRNA levels and hepatic iron stores (total and nonheme) (Fig 4C, insets). Furthermore, given that interleukin 6 (Il-6) and bone morphogenetic protein 6 (Bmp6) are regulators of hepatic *Hamp* expression, we also quantified the expression of these genes by qRT-PCR; both were generally lower in the LFe groups as compared to the others (data not shown). These data did not correlate with Hepc mRNA levels, however, so their significance is unclear.

### High-Iron Feeding Increased Tissue Iron Levels

Iron in bone (tibia) was increased in only the HFe groups (Table 8). Iron content of heart did not vary, while kidney iron levels were higher in only the HFe/AdCu and HFe/HCu groups. The iron content of isolated enterocytes [26] was higher in the HFe/LCu group; other differences were apparent, but due to large variation, they did not achieve statistical significance.

**Table 8:** Tissue Iron Levels.

μg/g	LFe/LCu	LFe/AdCu	LFe/HCu	AdFe/LCu	AdFe/AdCu	AdFe/HCu	HFe/LCu	HFe/AdCu		HFe/HCu
Serum	3.62 ± 2.97^a^* (8) ^≠^	5.29 ± 2.31^a^ (5)	7.22 ± 6.66^a^ (6)	24.05 ± 15.37^b^ (5)	14.14 ± 7.29^a^ (6)	8.17 ± 2.52^a^ (6)	10.94 ± 6.39^a^ (4)	10.81 ± 4.86^a^ (5)	25.16 ± 6.78^b^ (6)	
Heart	905 ± 226 (9)	745 ± 437 (6)	610 ± 66 (6)	1282 ± 897 (6)	860 ± 209 (6)	920 ± 365 (6)	620 ± 85 (6)	918 ± 436 (6)	1037 ± 503 (6)	
Kidney	357 ± 140^a^ (9)	368 ± 194^a^ (6)	260 ± 80^a^ (5)	403 ± 125^a^ (6)	586 ± 269^ab^ (6)	382 ± 47^a^ (6)	440 ± 197^a^ (6)	1065 ± 647^b^ (6)	1125 ± 201^c^ (6)	
IECs^†^	124 ± 73 (9)	94 ± 46 (6)	44 ± 48 (6)	113 ± 92 (6)	132 ± 140 (6)	143 ± 118 (6)	817 ± 926 (6)	438 ± 348 (6)	377 ± 350 (6)	
Bone	35 ± 15 (7)	24 ± 5.4 (4)	48 ± 38 (4)	53 ± 15 (4)	71 ± 15 (4)	54 ± 21 (4)	158 ± 32 (4)	168 ± 50 (4)	129 ± 17 (4)	

* Values are means ± SDs. Labeled means without a common letter differ, p<0.05 (2-factor ANOVA).

^≠^ Numbers in parentheses indicate n values.

^†^ Intestinal epithelial cells isolated from duodenum

### High-Iron Feeding Caused Systemic Copper Deficiency

**Fig 5:**
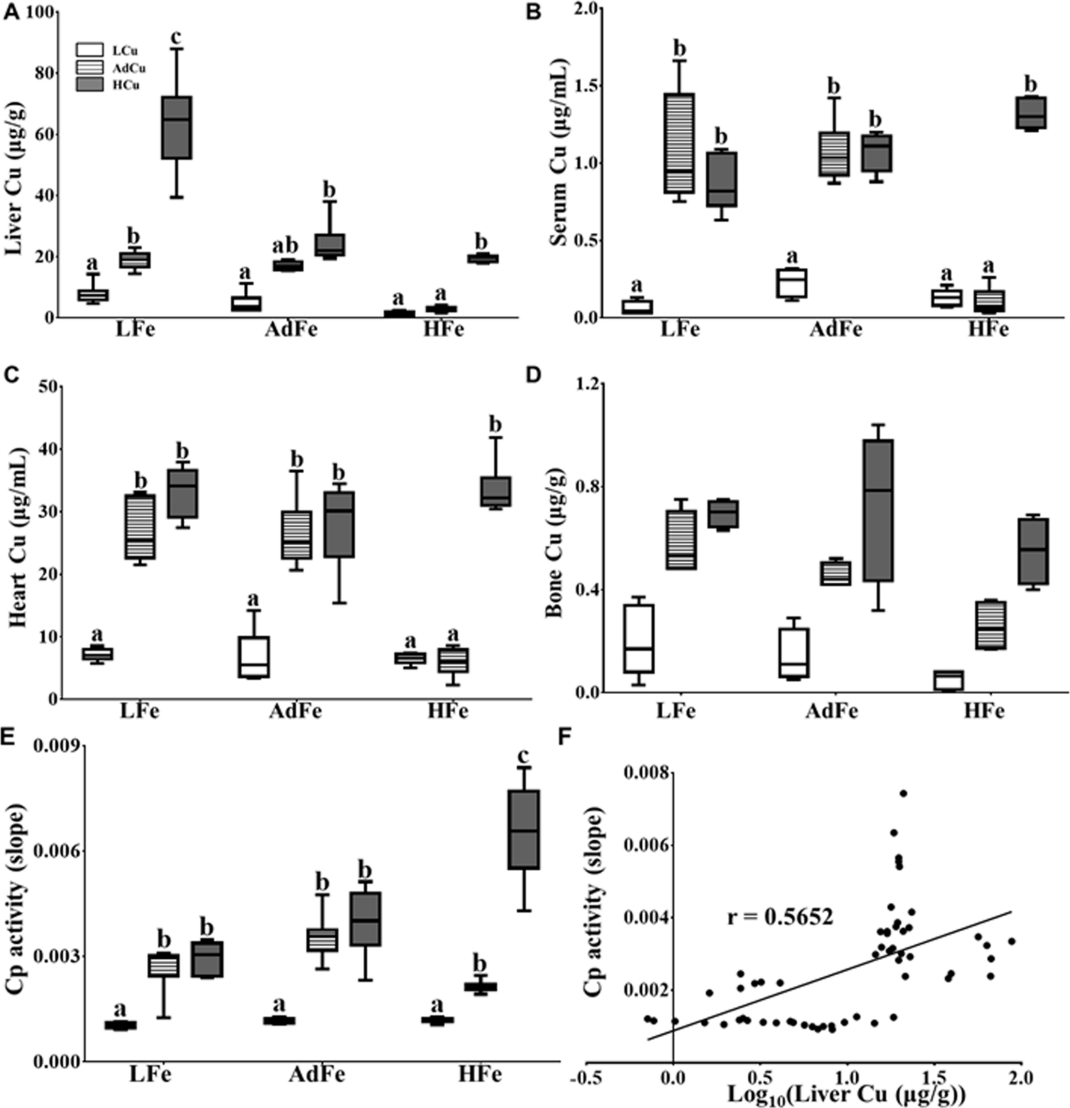
High-iron feeding resulted in severe tissue copper depletion and reduced Cp activity. The copper concentration in liver (**A**), serum (**B**), heart (**C**) and bone (**D**) was determined by ICP-MS. Cp (i.e. amine oxidase) activity was also measured in serum samples (**E**). The correlation between Cp activity and liver copper concentrations (log_10_) was calculated using Pearson product-moment correlation analysis (**F**). The line of best fit is shown along with the correlation coefficient (r) (*p*<0.0001). Labeled means without a common letter differ (*p*<0.05). n values and abbreviations used are the same as in Fig 1. The Box-and-Whisker plots indicate the following: the minimum value (the lower whisker), the lower quartile, the median, the upper quartile and the maximum value (the upper whisker). 2-way ANOVA factor analysis results are as follows: liver copper (Fe: *p*<0.0001; Cu: *p*<0.0001; Fe X Cu: *p*<0.0001); serum copper (Fe: *p*<0.05; Cu: *p*<0.0001; Fe X Cu: *p*<0.01); heart copper (Fe: *p*<0.0001; Cu: *p*<0.0001; Fe X Cu: *p*<0.0001); bone copper (Fe: *p*<0.01; Cu: *p*<0.0001; Fe X Cu: ns); and Cp activity (Fe: *p*<0.0001; Cu: *p*<0.0001; Fe X Cu: *p*<0.0001).

Results described above suggested that, predictably, rats consuming the low-iron diets developed iron-deficiency anemia (IDA). What was unexpected, however, was the development of anemia in rats consuming the HFe diets. Since anemia did not occur when the HFe diet contained extra copper, we postulated that decrements in Hb and Hct likely reflected copper-deficiency anemia (CDA). To directly address this possibility, we measured copper content in various tissues and blood as well as circulating levels of Cp, which is an accepted marker of severe copper deficiency [19, 27]. Liver copper content was lowest in the rats consuming the LCu diets, irrespective of iron content (Fig 5A). Hepatic copper content was also similarly diminished in the rats consuming the HFe/AdCu diet, but copper levels were similar to control levels in the HFe/HCu group. Moreover, significant hepatic copper loading occurred in the LFe/HCu group, which is consistent with previous observations that liver copper content increases in iron deficiency [28]. In general, this same pattern was also seen in regards to serum, heart and bone copper content (Fig 5B–5D). Furthermore, serum Cp (i.e. amine oxidase) activity was depressed in all LCu groups, with increasing copper content in the HFe diets leading to increments in Cp activity (Fig 5E). Cp activity correlated with liver copper content (Fig 5F), which supports the previous postulate that hepatic copper loading promotes biosynthesis of the *holo* (copper-containing) form of the Cp enzyme [10]. Moreover, kidney and enterocyte copper levels showed only minor variations with limited significance (Table 9). Overall, these data further support our postulate that the anemia caused by feeding a high-iron diet to weanling rats is the result of systemic copper deficiency.

**Table 9:** Tissue Copper Levels.

μg/g	LFe/LCu	LFe/AdCu	LFe/HCu	AdFe/LCu	AdFe/AdCu	AdFe/HCu	HFe/LCu	HFe/AdCu	HFe/HCu
Kidney	29.6 ± 16.1* (9) ^≠^	29.5 ± 5.5 (6)	34.7 ± 2.8 (6)	20.3 ± 3.1 (6)	30.5 ± 4.7 (6)	40.4 ± 9.9 (6)	12.4 ± 1.1 (6)	17.3 ± 5.3 (6)	30.8 ± 2.6 (6)
IECs^†^	0.60 ± 0.33^a^ (9)	2.23 ± 2.40^a^ (6)	1.34 ± 1.08^a^ (5)	1.08 ± 1.06^a^ (6)	0.71 ± 0.49^a^ (6)	6.69 ± 4.33^b^ (6)	1.01 ± 0.87^a^ (6)	0.90 ± 1.55^a^ (6)	4.16 ± 1.91^b^ (6)

* Values are means ± SDs. Labeled means without a common letter differ, p<0.05 (2-factor ANOVA).

^≠^ Numbers in parentheses indicate n values.

^†^ Intestinal epithelial cells isolated from duodenum.

## Discussion

This investigation tested the hypothesis that varying dietary copper intake would influence iron metabolism during disturbances of iron homeostasis. We chose a dietary approach in which groups of weanling rats were fed diets with low, normal or high iron content in combination with low, normal or high copper levels. As the feeding protocol proceeded, we noted that rats consuming the LFe diets grew slower than controls, as was anticipated. Unexpectedly, however, the same phenomenon was observed in the HFe groups, but decrements in growth were more severe. A reasonable postulate was that growth was impaired as a result of iron toxicity; however, final body weights of rats fed the high-iron diet with extra copper were not different from controls, suggesting that copper depletion was the underlying reason for growth deficits. It was also puzzling that impaired growth was not associated with reduced food consumption. However, considering the critical role of copper in energy metabolism, as a participant in electron transfer reactions in mitochondria (e.g. in cytochrome C oxidase), it was a logical postulate that impaired nutrient utilization (i.e. ATP synthesis) underlies growth defects. Growth deficits associated with copper deficiency have been reported previously [29, 30]. Other physiologic perturbations that were observed in rats consuming the HFe diets included: 1) cardiac hypertrophy, consistent with severe copper deficiency [31]; 2) anemia in the presence of adequate (or elevated) iron stores and normal serum nonheme iron levels; 3) robust induction of Epo in iron-replete animals; and 4) decreased tissue copper levels and reduced serum Cp activity. The fact that adding extra copper to the HFe diets prevented these physiologic perturbations proved that they were the consequence of copper depletion. High-iron feeding of rapidly growing rats thus causes CDA. Precedence for such mineral interactions has been established, as, for example, high zinc intake induces severe copper deficiency in humans [32, 33].

Our experimental findings support an earlier study which demonstrated that higher iron intake was associated with increased dietary copper requirements [34]. Moreover, previous investigations have provided evidence that iron, when in excess, can antagonize copper metabolism [35]. The mechanism by which high-iron feeding perturbs copper homeostasis occurs is unknown. One seemingly likely possibility is that high iron levels in the intestinal lumen impair copper absorption. To test this postulate, however, would require additional experimentation which is beyond the scope of the current investigation.

Other investigators have measured growth and Hb levels in rodents fed high-iron diets [36–38], including some studies that have used weanling SD rats [39, 40]. In general, most studies documented decrements in body weight after high-iron feeding. A mechanistic explanation for altered growth rates was, however, not provided. A few studies also measured Hb levels after high-iron feeding and showed no changes [36, 40] or an increase [39]. Differences between these previous studies and the current investigation could relate to the diets used, the length of feeding, the specific strain of mouse or rat used, and/or to rodent housing conditions.

In addition to the observation that high-iron feeding perturbs copper homeostasis, other notable findings have resulted from this investigation, all further supporting the concept that copper influences iron homeostasis. Although, in most cases, we cannot provide mechanistic explanations for these observations, they are nonetheless of potential physiologic significance. One interesting observation was that increasing the copper content of the HFe diets led to incremental increases in liver weights, with the HFe/HCu group having the largest livers (by mass) of all experimental groups. Hepatic Hepc expression and liver iron levels showed a similar pattern, with the highest Hepc mRNA expression and the greatest quantity of hepatic total and nonheme iron in the HFe/HCu group. Copper levels were normal in the livers of this group of rats. It is thus a plausible postulate that iron loading induced the noted hepatomegaly. How copper exacerbates liver iron accumulation in this dietary iron-overload model is unclear, but a reciprocal relationship between hepatic iron and copper content has been noted previously [1, 2]. Furthermore, higher copper in the high-iron diet caused an increase in nonheme serum iron (above the other HFe groups). This is consistent with increases in Tf saturation in rats consuming the HFe/HCu diet. Conceivably, higher Cp activity in these rats could increase serum iron levels by enhancing iron release from stores into the blood plasma. Moreover, higher copper intake in the iron-overload groups significantly reduced splenic nonheme iron levels. How this might occur is unclear, especially given that tissue and blood copper levels were normal (same as controls) in the HFe/HCu group.

We further noted that renal Epo mRNA expression and serum Epo protein levels were increased only in rats consuming the LFe/LCu and HFe/LCu diets. Given that Epo expression is induced by hypoxia (HIF signaling) [41, 42], it was surprising that renal Epo levels were not increased in the LFe/AdCu and LFe/HCu groups, as rats consuming these diets were anemic (and thus also likely hypoxic). The main difference between the anemic/hypoxic rats that showed robust Epo expression and those that showed lesser or no induction was thus dietary copper deprivation. Since copper deficiency causes anemia, it is a logical postulate that copper deprivation can induce Epo expression; we did indeed find one published report showing a similar phenomenon [43]. It thus appears that CDA is a stronger driver of renal Epo expression than iron deprivation. Elucidating the mechanism by which this occurs is an experimental imperative for future investigation.

Another notable observation deriving from this investigation relates to hepatic Hepc mRNA expression. The *Hamp* gene is *trans*-activated when body iron stores increase. One mechanism by which this occurs relates to competition between transferrin receptors expressed in hepatocytes (TfR1 and TfR2) [44, 45]. When serum Tf saturation levels are high, TfR2 out competes TfR1 for available diferric Tf, thus inducing an intracellular signaling cascade that increases *Hamp* gene transcription. In our investigation, we noted that high-iron feeding led to induction of Hepc mRNA levels, as anticipated. What was unexpected, however, was that higher copper intake further increased Hepc mRNA levels in some iron-loaded rats, suggesting that copper may influence *Hamp* expression.

When body iron stores are low and erythropoietic demand increases (due to anemia/hypoxia), the *Hamp* gene is effectively silenced. A recently discovered peptide hormone, called erythroferrone (Erfe) [22], produced and secreted by developing erythrocytes and the spleen, has been proposed as an erythroid regulator of iron homeostasis. It was further suggested that Epo induces Erfe expression, and that Erfe then downregulates hepatic *Hamp* expression, thus allowing robust intestinal iron absorption and iron release from stores. Since Erfe transcript levels increase in response to erythropoietic stress, we quantified Erfe mRNA expression levels in the spleens of our experimental rats. As described above, we noted robust Epo expression in only 2 groups of rats, those consuming the LFe/LCu and the HFe/LCu diets. We thus expected that Erfe expression would be increased in these groups. This prediction was correct in regards to the rats consuming the LFe/LCu diets, but conversely, Erfe expression was very low in the rats consuming the HFe/LCu diet. This is consistent with hepatic Hepc mRNA levels in these groups (i.e. low in the LFe/LCu group and much higher in the HFe/LCu group), since Erfe is proposed to downregulate *Hamp* expression. To understand why Erfe would be differentially expressed in these dietary groups, in spite of significant anemia and robust Epo expression in both, it is necessary to identify differences in the pathological phenotypes. Further, since Erfe transcript levels did not increase in response to circulating Epo in the HFe/LCu group, it is logical to consider changes in the spleen. Notably, splenic nonheme iron was low in the LFe/LCu group but >45 times higher in the HFe/LCu group. It could thus be that high splenic iron inhibits Epo-induced Erfe expression. The physiologic signals associated with high systemic iron in the HFe/LCu group may trump the anemia (which relates to low copper in these rats), so Erfe expression remains low and Hepc expression remains high.

In summary, this investigation has revealed heretofore unrecognized interactions between the essential trace minerals iron and copper. High-iron feeding with low or adequate copper levels was shown to induce CDA in growing rats. Although the mechanism by which copper deficiency causes anemia is unknown, it likely relates to an unidentified copper-dependent step in mitochondrial heme synthesis in developing erythrocytes [2]. The phenotype of CDA in rats may be more severe than that associated with IDA, as exemplified by the more significant growth retardation seen in the copper-deprived rats (but iron toxicity may have also contributed). Adding extra copper to the HFe diet prevented the development of CDA, essentially proving that the noted physiologic perturbations directly related to copper. These findings raise the question of whether iron supplementation in humans could, over the long term, induce deficiencies in copper, as has been proposed before [34, 35]. Although this investigation used a very high level of iron, there are examples of humans who, for medical reasons, consume large quantities of iron. For example, patients with chronic kidney disease are often treated with phosphate binders [46], since hyperphosphatemia is common in this condition [47]. One such phosphate binder is ferric citrate [48, 49]. Patients with end-stage disease (stage 4 or 5) may thus receive 0.21 grams of iron up to 3 times per day (as ferric citrate) for long periods of time [50]. This is up to >35 times more iron than the typical human consumes from a normal varied diet (average ~18 mg/day). Other groups which are likely to require iron supplementation are pregnant women, women of childbearing age, those chronically consuming proton-pump inhibitors for gastric acid reflux, and those suffering from malabsorptive disorders (e.g. Crohn’s disease, colitis) or after gastric bypass surgery. An interesting question relates to whether extra copper should be added to iron supplements to avoid any untoward effects of high iron intake on copper homeostasis. This concept was proposed earlier [34] and the current work provides additional support for this contention. Interestingly, iron supplements containing extra copper were promoted for the anemia of pregnancy in the 1930s, but their use seems to have ended sometime shortly thereafter [35]. One future goal is to define the minimum amount of dietary iron that is required to induce copper deficiency in rats, so as to be able to better extrapolate results to humans who consume iron supplements.
